# Testing for causality between systematically identified risk factors and glioma: a Mendelian randomization study

**DOI:** 10.1186/s12885-020-06967-2

**Published:** 2020-06-03

**Authors:** A. E. Howell, J. W. Robinson, R. E. Wootton, A. McAleenan, S. Tsavachidis, Q. T. Ostrom, M. Bondy, G. Armstrong, C. Relton, P. Haycock, R. M. Martin, J. Zheng, K. M. Kurian

**Affiliations:** 1 Brain Tumour Research Centre, Institute of Clinical Neurosciences, University of Bristol, Bristol, UK; 2 MRC Integrative Epidemiology Unit (IEU), Bristol Medical School, University of Bristol, Oakfield House, Oakfield Grove, Bristol, BS8 2BN UK; 3 School of Psychological Science, University of Bristol, Bristol, UK; 4 NIHR Biomedical Research Centre at the University Hospitals Bristol NHS Foundation Trust and the University of Bristol, Bristol, BS8 2BN UK; 5 Population Health Sciences, Bristol Medical School, University of Bristol, Bristol, UK; 6 Section of Epidemiology and Population Sciences, Department of Medicine, Baylor College of Medicine, Houston, TX UK; 7 The National Institute for Health Research Bristol Biomedical Research Centre, University Hospitals Bristol NHS Foundation Trust and University of Bristol, Oakfield House, Oakfield Grove, Bristol, BS8 2BN UK

**Keywords:** Mendelian randomization, Glioma, Risk factor, Systematic search, Causal inference

## Abstract

**Background:**

Whilst epidemiological studies have provided evidence of associations between certain risk factors and glioma onset, inferring causality has proven challenging. Using Mendelian randomization (MR), we assessed whether associations of 36 reported glioma risk factors showed evidence of a causal relationship.

**Methods:**

We performed a systematic search of MEDLINE from inception to October 2018 to identify candidate risk factors and conducted a meta-analysis of two glioma genome-wide association studies (5739 cases and 5501 controls) to form our exposure and outcome datasets. MR analyses were performed using genetic variants to proxy for candidate risk factors. We investigated whether risk factors differed by subtype diagnosis (either glioblastoma (*n* = 3112) or non-glioblastoma (*n* = 2411)). MR estimates for each risk factor were determined using multiplicative random effects inverse-variance weighting (IVW). Sensitivity analyses investigated potential pleiotropy using MR-Egger regression, the weighted median estimator, and the mode-based estimator. To increase power, trait-specific polygenic risk scores were used to test the association of a genetically predicated increase in each risk factor with glioma onset.

**Results:**

Our systematic search identified 36 risk factors that could be proxied using genetic variants. Using MR, we found evidence that four genetically predicted traits increased risk of glioma, glioblastoma or non-glioblastoma: longer leukocyte telomere length, liability to allergic disease, increased alcohol consumption and liability to childhood extreme obesity (> 3 standard deviations from the mean). Two traits decreased risk of non-glioblastoma cancers: increased low-density lipoprotein cholesterol (LDLc) and triglyceride levels. Our findings were similar across sensitivity analyses that made allowance for pleiotropy (genetic confounding).

**Conclusions:**

Our comprehensive investigation provides evidence of a causal link between both genetically predicted leukocyte telomere length, allergic disease, alcohol consumption, childhood extreme obesity, and LDLc and triglyceride levels, and glioma. The findings from our study warrant further research to uncover mechanisms that implicate these traits in glioma onset.

## Background

Glioma is a rare cancer with age adjusted incidence rates range from 4.67 to 5.73 per 100,000 [1, 2]. Despite this, brain tumours such as glioma cause the greatest number of years lost to cancer to those under 40 years of age [3]. The health burden posed by glioma is due to its poor prognosis, with an overall 5-year survival rate of under 20% and significant morbidity in survivors [4–6]. While there have been efforts to identify risk factors for glioma, evidence has been inconsistent [7–22] and the aetiology of glioma remains largely unclear [4].

Mendelian randomization (MR) is a method to appraise causality within observational epidemiology. It utilizes germline genetic variants that are robustly associated with potentially modifiable exposures as proxies (‘instrumental variables’ [IVs]) for the risk factor of interest [23]. As germline genetic variants tend to be randomly distributed with respect to most human traits in the general population [24], MR studies are less likely to be affected by the sorts of confounding factors that typically bias observational findings [25, 26]. Additionally, as germline genotypes cannot be affected by the presence of disease, the generation of spurious results through reverse causation is avoided [27]. Germline genetic variants can therefore be regarded as randomised proxies for an exposure of interest, in the same way that the allocation group in a randomised controlled trial (RCT) is a proxy for an intervention of interest [28]. MR studies can prioritise targets for further research or for intervention development in an RCT, and may provide more reliable findings than conventional epidemiology to help inform public health policies when an RCT is not possible [28].

MR analysis is based upon the following three assumptions (Additional file Figure 1) [28]: the single nucleotide polymorphisms (SNPs) selected as IVs to proxy the exposure are robustly associated with the exposure; the SNPs have no relationship with any confounders of the exposure–outcome association; and the SNPs are only associated with the outcome through their effect on the exposure. Within the constraints of these assumptions, SNPs can be used as proxies for a large range of modifiable exposures. Two-sample MR techniques allow analysis using summary data from genome wide association studies (GWAS) conducted in two independent samples: one set for the exposure of interest and one for the outcome [29]. An important application of MR is to elicit causal evidence for putative observational associations in cancer [30].

There have been previous MR studies that have investigated potential risk factors for glioma. One such study implicated genetically predicted increases in telomere length were associated with an increased risk of glioma [31, 32]. Other conventional observational studies have shown negative results for risk factors, such as for obesity-related factors, vitamin D and atopy [33–35].

The aim of this study was to identify risk factors that have been investigated using traditional observational epidemiology and to examine the causal nature of the association between these putative risk factors and glioma onset. Glioma is a highly heterogeneous disease, with varying genetic profiles both intra- and inter-tumourally [36]. Therefore, we conducted subtype analyses by splitting the outcome data into glioblastoma or non-glioblastoma (low grade glioma) cases only. To increase statistical power, our main analyses used the full outcome data regardless of subtype diagnosis (consisting of glioblastoma and non-glioblastoma cases). Putative associations were then evaluated using a two-sample MR approach using glioma summary data from a recent GWAS [37] meta-analysis.

## Methods

We conducted a GWAS meta-analysis of glioma and a two-sample MR analysis using summary GWAS data. Ethical approval was not required for this specific analysis as the entirety of the data was sourced from the summary statistics of a published GWAS and no individual-level data were used. A summary of the analysis plan can be found in Additional file Figure 2.

### Genetic instrument selection

To systematically and comprehensively identify all previously reported non-genetic or epigenetic risk factors for glioma from the existing published literature, we conducted a formal systematic search of MEDLINE from inception to October 2018 using the Ovid Platform [38]. Details of the search strategy and inclusion criteria are provided (Additional file Note). To ensure the same text was not screened multiple times, duplicates were removed using the duplicate removal function in Endnote X_7_ software. All studies were then screened based on title and abstract by the lead author. If the study was included at this stage the full text was retrieved and reviewed for eligibility by the lead author. Risk factors from eligible studies were extracted. No results (association between risk factor and glioma) were extracted from these studies as our interest was identifying putative risk factors for subsequent MR analysis, not summarizing the results.

The summary genetic instrumental variables for the 36 identified risk factors were primarily collated from GWAS, details of which are given in Additional file Table 1. Where the full GWAS results were not available, the instruments were collated from the NHGRI-EBI GWAS Catalogue [39]; alternatively when the full summary results were available, instruments were collated from MR-Base [40]. Genetic instruments were formed using SNPs shown to robustly (*P* < 5 × 10^− 8^) and independently (*r*^2^ < 0.001) associated with the risk factor under examination in individuals of European ancestry.

To undertake the MR analysis, we gathered the following parameters from the summary results: the regression coefficient (e.g. beta or log odds ratio) quantifying the association of each SNP with the exposure of interest from an additive genetic model; the standard error of the regression coefficient; the effect allele; non-effect allele; and the effect allele frequency. The effect allele was the allele that was related to an increased odds/levels of the exposure (Additional file Table 1).

To instrument allergic disease, we used a shared genetic instrument of broad allergic disease that considered the presence of asthma, hay fever or eczema. We chose to instrument allergic disease using a shared genetic instrument due to a shared genetic origin that results in the coexistence of these atopic disorders [41, 42].

### Genetic associations of glioma via GWAS meta-analysis

The second stage of the analysis involved the collection of the outcome data: i.e. the relevant summary genetic data from a glioma GWAS. These summary data were obtained from the principal investigators of a glioma GWAS consortia and relate to 5739 cases and 5501 controls from two independent GWAS studies of European ancestry [43, 44]. To determine whether the risk factors differ between subtypes we considered glioma as being either glioblastoma (3112 cases, 5501 controls) or non-glioblastoma (2411 cases, 5501 controls). The GWAS information was provided as summary data from the two different consortia: Glioma International case-control study (GICC) which is comprised of 4564 cases and 3256 controls; and the University of Texas M.D. Anderson Cancer center (MDA) which included 1175 cases and 2236 controls. The individual GWAS were adjusted for sex, age and the first two principal components (to reduce the likelihood of confounding via population stratification). Individual studies have restricted statistical power to detect precise effect estimates. Thus, to gain a more complete understanding of glioma risk, we performed a meta-analysis of these two previously published GWAS [43, 44]. We also performed a glioblastoma meta-analysis and non-glioblastoma meta-analysis. GICC provided 2460 glioblastoma cases and 3265 controls and MDA provided 652 glioblastoma cases and 2236 controls. For non-glioblastoma, GICC comprised of 1898 cases and 3265 controls and MDA 513 cases and 2236 controls. Meta-analyses were implemented using the fixed-effects inverse-variance method, based upon the β effect estimates and standard errors from each consortium using METAL (metal-2011-3-25) [45].

LD score regression was used to check the quality of our meta-analysis by evaluating the degree of genomic inflation in the glioma GWAS due latent sources of bias [46, 47]. LD scores were calculated from the meta-analysis. In order to gain aetiological insights, the genetic correlation between the MR top findings and glioma were computed and SNP heritability (the amount of variation in a trait that is attributable to genetic factors [48]) for the glioma, glioblastoma and non-glioblastoma datasets was also calculated.

### Two-sample MR analysis

We systematically explored the causal relationship of the identified risk factors that could be proxied using genetic instruments on glioma using a multiplicative random effects inverse-variance weighting (IVW) approach in two sample MR. Horizontal pleiotropy is a major source of confounding in MR studies [49, 50], so to minimise this we performed sensitivity analyses using the weighted median estimator (WME), the mode-based estimator (MBE) and MR-Egger regression [51–53]. A consistent effect across the multiple methods would give us the strongest evidence for a causal effect and suggest that our results are not biased by horizontal pleiotropy. Random effects IVW assumes that if the causal estimates due to each SNP differ, these deviations are equal [40]. The WME requires at least half of the genetic information to be derived from valid instrumental SNPs, with stronger SNPs contributing more to the estimate, for the causal estimate to be unbiased [54]. The MBE clusters SNPs into groups determined by their similarity of causal effects and returns the causal effect estimate based on the cluster that has the greatest number of SNPs [40], giving an unbiased estimate if the SNPs in the largest cluster are valid, even if most SNPs are invalid instruments. To further assess the impact of horizontal pleiotropy we performed MR-Egger regression, a type of MR analysis that can quantify the amount of bias caused by directional pleiotropy (when the average value of the pleiotropy distribution is not balanced i.e. non-zero [51]) based upon the intercept from this analysis [55, 56]. MR-Egger regression provides an unbiased effect-estimate even if all the SNPs are subject to horizontal pleiotropy, although it requires the InSIDE (instrument strength independent of direct effects) assumption to be valid. MR-Egger regression also requires a large number of instrumental SNPs otherwise the method is underpowered. Furthermore, to examine the effect of SNP outliers in the MR analysis, we undertook a leave-one-out analysis which removes one SNP at a time and re-calculates the association results [55]. To assess evidence for heterogeneity, a potential indicator of horizontal pleiotropy, we used Cochran’s Q statistic [57] and Rucker’s Q test [58]. In cases where there was evidence for heterogeneity, results were further assessed through Radial plots, which provide improved visualisation of outliers [59].

To increase the likelihood that MR infers the correct causal direction between an exposure and glioma we applied directional (Steiger) MR to test for reverse causation [60]. This calculates the variance explained by the SNPs that form the exposure and outcome data and compares these to estimate whether the direction of effect is orientated from exposure to outcome or vice versa.

For the non-binary exposures, MR results are reported as odds ratios (OR) (95% confidence intervals (CI)) per 1 standard deviation (SD) change in each genetically predicted risk factor. For the binary risk factors, the OR were converted (by raising the OR and 95% CI by 0.693) to represent the OR per doubling in the odds of the risk factor [61].

### Polygenic risk score analysis to improve statistical power

Polygenic risk scores (PRS) can be used to assess putative causal associations [28, 62] (see Additional file Figure 3). PRS use a less stringent *P* value threshold for inclusion of SNPs (*P* < 1 × 10^− 5^) and thus increase power as they capture more trait variance due to a greater number of genetic variants being included. However, they assume no horizontal pleiotropy and thus are more susceptible to false positive associations [63]. The PRS is equivalent to an MR analysis using a fixed effects IVW model. To potentially enhance detection of causal associations we applied BADGERS (Biobank-wide Association Discovery using Genetic Risk Scores) to examine associations between the instrumental risk factors, where the full GWAS summary data were available, and glioma onset using GWAS summary statistics [64].

Of the 36 instrumental risk factors, the full GWAS summary results were available for 30 traits. PRS were derived using independent SNPs for each GWAS (*P* < 1 × 10^− 5^) based on r^2^ < 0.001 using genotype data from European individuals (CEU) from phase 3 (version 5) of the 1000 Genomes project. PRS were constructed using the risk factor GWAS data and the glioma meta-analysis as the outcome.

### Power estimation

We performed *post-hoc* power calculations based on a method provided by Burgess [65] (see Additional file Table 2). Power calculations were performed using effect estimates from the MR analysis to ascertain whether we had adequate sample size to detect the MR point estimate per SD change in genetically increase in each non-binary risk factor (α assumed to be 0.05).

### Interpretation of results

We analysed the association of 36 genetically instrumented risk factors with glioma. We imposed a Bonferroni-corrected significance level to determine statistically significant results of *P* < 1 × 10^− 3^ (0.05 / 36, the amount of risk factors included in our analysis) and a suggestive threshold of 1 × 10^− 3^ ≤ *P* < 0.05. All MR analyses were performed using the Two-Sample MR package in R [40].

## Results

### Risk factor selection

**Fig. 1:**
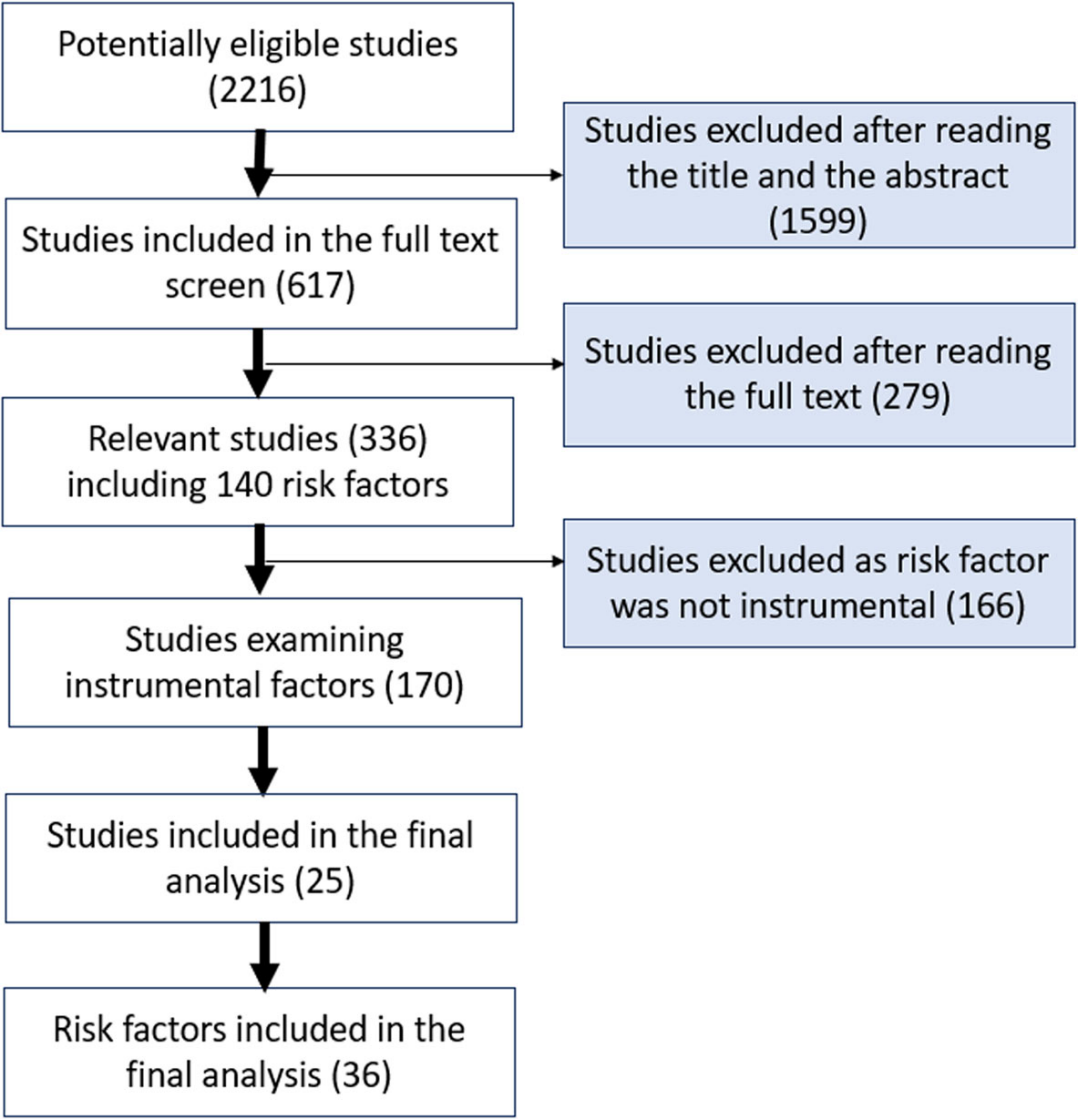
Flow diagram of risk factor inclusion

Of the 170 studies examining instrumental glioma risk factors, there were 36 unique risk factors that had suitable genetic variants available for instrumentation. Figure 1 summarizes the screening process which resulted in the inclusion of 25 studies (Additional file Table 3) investigating 36 risk factors. Additional file Note 2 summarizes all the risk factors that were identified in the systematic search before exclusion due to lack of instrumentation.

### Genetic arichitecture of glioma

Univariate LD score regression suggested that the 1,201,423 common variants we included in the meta-analysis explained 2.6% of the phenotypic variance of glioma risk (H^2^ = 0.0257, S.E. = 0.0425); 1,201,269 SNPs explained 1.1% of the phenotypic variance of glioblastoma risk (H^2^ = 0.0115, S.E. = 0.0537); and 1,201,154 SNPs explained 9.2% of the phenotypic variance of non-glioblastoma risk (H^2^ = 0.0928, S.E. = 0.0599). Due to the limited sample size, genetic correlation between glioblastoma and non-glioblastoma tumours could not be estimated.

For our GWAS meta-analysis, there was little evidence to suggest inflation of results for glioma, glioblastoma and non-glioblastoma. The genomic inflation factor *λ*GC was 1.0345 and the LD score regression intercept 1.045 for glioma; *λ*GC was 1.0315 and the LD score regression intercept 1.0398 for glioblastoma; and *λ*GC was 1.0165 and the LD score regression intercept 1.0133 for non-glioblastoma.

### Two-sample MR to investigate putative associations with glioma

**Fig. 2:**
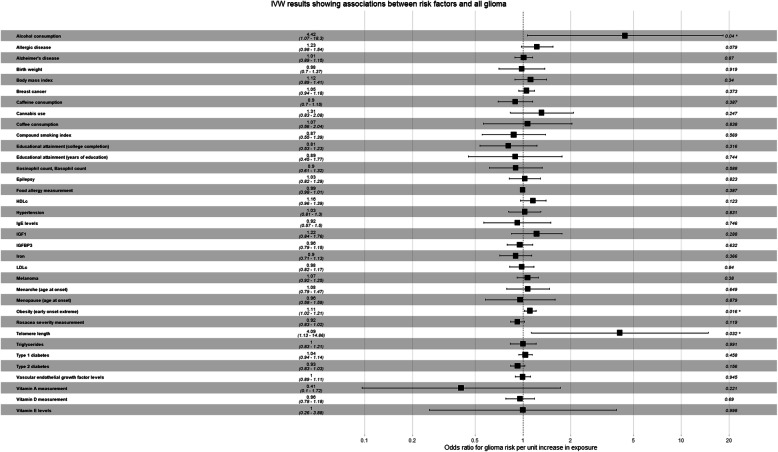
Inverse-variance weighted estimates for the association between genetically increased risk factors and odds of glioma. LDLc refers to low density lipoprotein cholesterol and HDLc to high density lipoprotein cholesterol. The ‘lifetime smoking index’ measure, combines multiple smoking behaviours (smoking initiation, smoking duration, smoking heaviness, and smoking cessation)

Full results for the IVW MR analysis of putative risk factors are presented for: glioma (Fig. 2), glioblastoma (Additional file Figure 4) and non-glioblastoma (Additional file Figure 5). A list of risk factors that met at least the suggestive *P* value threshold for any subtype diagnosis are given in Table 1. In short, none of the putative risk factors reached the strict *P* value threshold but six risk factors did meet the weaker threshold for suggestive evidence: telomere length (risk factor for all glioma and non-GBM), alcohol consuption (risk factor for all glioma and GBM), childhood extreme obesity (risk factor for all glioma and GBM), LDLc levels (protective factor for non-GBM), allergic disease (risk factor for GBM) and trigylcerides levels (protective factor for non-GBM).

**Table 1:** Associations from the IVW analysis that met at least the suggestive *P* value threshold. OR, CI and *P* value are from the IVW analysis. Results from the three sensitivity analyses (MR Egger, WME and MBE) are given in brief and take three values: “agree”, *P* value meets at least 0.05 and direction and magnitude of effect agrees with the IVW results; “disagree”, *P* value meets at least 0.05 and direction or magnitude of effect does not agree with the IVW results; “uncertain”, when the *P* value does not meet significance. Heterogeneity results are from the MR Egger intercept: high values of heterogeneity indicates potential pleiotropy. The instrument for telomere length did not consist of enough SNPs to conduct this analysis. Full results are given in detail in Additional file Table 4

Risk Factor	Subtype	OR	95% CI	*P* Value	MR Egger	WME	MBE	Heterogeneity (*P* value)
Telomere length	Non-GBM	4.05	1.72 to 9.56	1.38 × 10^−3^	Uncertain	Agree	Agree	NA
Alcohol consumption	GBM	8.37	1.69 to 41.54	9.36 × 10^−3^	Uncertain	Agree	Uncertain	0.43 (8.08 × 10^−1^)
Obesity (childhood extreme)	All glioma	1.11	1.02 to 1.21	1.63 × 10^−2^	Uncertain	Uncertain	Uncertain	5.19 (2.68 × 10^−1^)
Obesity (childhood extreme)	GBM	1.12	1.02 to 1.22	2.07 × 10^−2^	Uncertain	Uncertain	Uncertain	2.56 (6.34 × 10^−1^)
Telomere length	All glioma	4.09	1.13 to 14.86	3.24 × 10^−2^	Uncertain	Agree	Uncertain	NA
LDLc	Non-GBM	0.79	0.63 to 0.99	3.99 × 10^−2^	Uncertain	Uncertain	Uncertain	76.71 (5.20 × 10^−1^)
Alcohol consumption	All glioma	4.42	1.07 to 18.30	4.05 × 10^−2^	Uncertain	Agree	Uncertain	0.05 (9.75 × 10^−1^)
Allergic disease	GBM	1.29	1.01 to 1.67	4.76 × 10^−2^	Uncertain	Uncertain	Uncertain	185.58 (1.86 × 10^−9^)
Triglycerides	Non-GBM	0.77	0.59 to 1.00	4.86 × 10^−2^	Agree	Uncertain	Uncertain	77.49 (2.52 × 10^−1^)

### Sensitivity analyses of MR findings

Risk factors that met at least the suggestive *P* value threshold and were associated with any subtype were eligible for follow up sensitivity analyses.

The results from the MR-Egger analysis mostly did not reach statistical significance except in the case of trigylcerides and non-GBM (OR_non-GBM_ = 0.65, 95% CI_non-GBM_ = 0.46 to 0.91, *P*_non-GBM_ = 1.2 × 10^− 2^), which agrees with the direction of effect from the IVW analysis. The MR-Egger method hightlights the presence of pleiotropy in only allergic disease for GBM (intercept = 185.58, *P*_intercept_ = 1.86 × 10^− 9^). The MR-Egger analysis for alcohol has large confidence intervals due to a lack of power (four SNPs) indicating the need for larger sample sizes and better powered analyses.

We compared the results from the MBE and WME methods against the IVW as a further sensitivity test. These tests were only significant in the cases of telomere length in both all glioma and non-GBM and alcohol consumption in all glioma and GBM. These results are summarised in Table 1 and fully in Additional file Table 4.

We performed Cochran’s Q test on our instruments to test for heterogeneity. This test indicated hetereogeneity was present in the allergic disease-GBM association (Q = 187.49, *P* = 2.44 × 10^− 9^) which could suggest a violation of the third MR assumption (that is, SNPs are only associated with the outcome through their effect on the exposure).

We used radial IVW to construct radial IVW regression estimates and lists of outlier SNPs with high heterogeneity. Only three associations could be tested this way: LDLc and non-GBM, which included the null; allergic disease and GBM, which agreed with the IVW result; and triglycerides and non-GBM, which included the null. These results are presented in Additional file Table 4.

We also implemented directionality (Steiger) test to estimate the orientation of the direction of effect. In brief, all of the associations that met at least the suggestive threshold showed the correct orientation of effect (i.e. from exposure to outcome); more in-depth results are presented in Additional file Table 4.

### Polygenic risk score associations

Two traits were associated with all glioma: melanoma (*P*_all glioma_ = 2.12 × 10^− 3^) and allergic disease (*P*_all glioma_ = 1.20 × 10^− 2^). All other traits did not meet the significance threshold. Full results are given in Additional file Table 5.

Similar results were seen for glioblastoma (melanoma, *P*_glioblastoma_ = 2.50 × 10^− 3^; and allergic disease, *P*_globlastoma_ = 1.41 × 10^− 2^). For non-glioblastoma, we observed only a positive correlation for melanoma (*P*_non-glioblastoma_ = 1.51 × 10^− 2^). Full results are given for glioblastoma in Additional file Table 6 and for non-glioblastoma in Additional file Table 7.

## Discussion

This study has systematically identified all published hypothesised glioma risk factors and applied these to a rigorous statistical framework that investigated the causal relationships between the instrumented candidate risk factors and glioma incidence. The results from this approach support previous existing evidence that genetically predicted longer telomeres are associated with increased glioma risk. Additionally, we find suggestive evidence that the following genetically predicted traits are risk factors for glioma: increased alcohol consumption increases risk; childhood extreme obesity increases risk; and increased levels of LDLc and triglycerides decrease risk of low-grade glioma (non-glioblastoma). Our analysis highlighted little causal evidence for other risk factors reported in the literature.

The evidence we found to support a link between genetically predicted leukocyte telomere length and glioma risk come from both the PRS we conducted as well as the MR study. These methods suggest that longer telomeres, as predicted by genetic proxies, increase risk of glioma, supporting previous MR studies (cases = 1130) and also a case-control study (cases = 467) [31, 32, 66]. It is important to note that our results do not suggest that measured longer telomeres associate with increased glioma risk but only genetically predicted longer telomeres. The consistency across studies highlights the robustness of our result as we used independent glioma GWAS data to the previous MR studies. The implications of genetically predicted longer telomere length and glioma risk are discussed in detail in Haycock et al. (2017) [31]. In short, telomere shortening is thought to act a tumour suppressor, restricting the proliferation of neural stem cells. Individuals with longer telomere have greater proliferative potential and therefore may be more likely to acquire somatic mutations [31, 67]. This seemingly paradoxical link between telomere length and carcinogenesis, whereby longer telomeres, instead of shorter telomeres, lead to an increased risk of cancer, has yet to be fully elucidated. Aviv, et al. in their article describe a potential solution to this paradox based on existing evidence, though further research is necessary to fully explore this [68].

We found that a genetic liability to allergic disease increases risk of glioblastoma, contrary to previous epidemiological evidence which supports an inverse relation to risk [69]. The concern is whether the underlying relationship is causal as the majority of support has been derived from case-control studies [70] which are liable to recall bias due to cognitive impairment [70]. A prospective cohort-based analysis did not find strong evidence that atopy protects against glioma [71]. A possible explanation for the conflicting findings for the relationship between allergic disease and glioma is due to reverse causation in such studies – with the presence of glioma causing people to under-report co-morbidities – resulting in the spurious generation of an inverse association. This is supported by the fact that glioblastoma is known to cause immunosuppression [72] and therefore may result in a reduction of atopy expression in glioma patients, making it appear as if atopy protects against glioma. To limit the potential of reverse causation as an explanation of our MR results, we conducted directional (Steiger) MR and found little evidence that glioma is driving the MR associations we observed. Furthermore, there is a real difference between genetic liability to allergic disease, which we have investigated, and actual presence of allergies, which would be commonly studied in observational research. An individual may be prone to an allergic disease due to their genes, but not develop one; conversely, environmental factors may produce allergies in those with no genetic liability at all. This distinction is key to make and will help further research into the true link between allergies, atopy, and glioma risk. Furthermore, for the MR analysis, we used an updated atopy instrument from a recent GWAS that identified shared genetic variants of allergic disease. Common involvement of inflammatory pathways might speculatively reflect the mechanism by which allergic disease contributes to glioma risk; however, further research is needed to investigate this.

We also found three metabolic-related traits were associated with glioma: genetically predicted childhood extreme obesity increases risk for all glioma and GBM, and genetically predicted LDLc and triglyceride levels decrease risk for non-GBM. Metabolic traits such as these generally have a high level of interplay that makes disentangling true causality difficult to ascertain. Traits such as these have been implicated heavily in meningioma – particularly obesity – but within glioma their effects are less certain. Particularly interesting is the difference between subtype diagnosis and how LDLc and triglyceride levels appear only in non-GBM but not in all glioma or GBM. These traits would require further follow-up to ascertain their true causal nature in glioma risk.

Furthermore, genetically predicted alcohol consumption was demonstrated to increase risk in all glioma and glioblastoma. However, the confidence intervals from these analyses are very wide and the alcohol SNPs used as IVs in our analysis only explained 0.1% of the variance in alcohol consumption. Several observational cohort and case-control studies have provided evidence that alcohol consumption is positively associated with glioma risk, but the direction and magnitude of this relationship remains controversial [73–80]. Several potential mechanisms have been suggested to explain the relationship between alcohol consumption and glioma risk [75]. These proposed mechanisms are speculated to be a consequence of the products of alcohol metabolism [81]. Both the MR point estimate and PRS indicated a positive link between alcohol consumption and glioblastoma. However, the wide confidence intervals obtained reflect the underpowered nature of this sub-group analysis and the low precision of the estimates. Further analysis with larger sample sizes and additional alcohol genetic instruments is required to disentangle causality.

Important within our study is the subtype analyses we conducted (looking at all glioma, glioblastoma and non-glioblastoma) due to the extremely heterogeneous nature of the disease both intra- and inter-tumourally. It has been shown extensively that glioblastoma exhibits a different genetic profile and variation from lower grade tumours [82]. However, we still chose to conduct an all glioma analysis, which included any case regardless of subtype, due to the increase in statistical power gained from doing so. Similarly, a caveat to subtype analyses such as this is the loss of statistical power due to lower sample sizes in an already rare cancer. This resulted in particularly wide confidence intervals in our MR results and thus limited robust interpretation of the results. It is highly likely that glioblastoma and low-grade glioma have different aetiological drivers that our analysis could not pick up due to lack of power. Further research with larger glioblastoma and non-glioblastoma GWAS datasets is required to investigate this further.

The results from the PRS analysis indicated that individuals with a genetic liability to melanoma have increased odds of developing glioma, glioblastoma and non-glioblastoma. But, in the MR analysis we found little to suggest an association between melanoma and glioma. As a method, PRS is susceptible to a high rate of false positives due to the presence of horizontal pleiotropy despite increasing statistical power of complementary methods [63]. Thus, further robust analyses are required to investigate this result.

Strengths of this analysis include the systematic identification of hypothesized risk factors and the inclusion of 36 instrumental candidate risk factors for glioma; the inclusion of summary data for the risk factors from GWAS with large sample sizes (to infer reliable causal effect estimates); and the use of an MR framework. An advantage of the MR approach is that by using germline genetics variants as proxies for exposures, bias caused by reverse causation is avoided, as well as, a reduction in bias caused by confounding. We implemented PRS to help improve the power of our analysis and further validate the direction of effect of our findings.

A limitation of Mendelian randomisation is that it cannot distinguish between the different types of pleiotropy. There are two types of pleiotropy: vertical, where the instrument affects the outcome through the pathway of the exposure; and horizontal, where the instrument affects the outcome via a different pathway, bypassing the exposure of interest. This means that causality, itself a form of vertical pleiotropy, cannot be accurately separated from horizontal pleiotropy. This confounding is hard to address without extensive knowledge of the underlying biological systems and mechanisms, which are oftentimes unknown. However, the presence of horizontal pleiotropy can be examined using sensitivity analyses, the like of which we have employed in our research, such as with the MR-Egger method. This method is still not perfect, however, due to instruments requiring at least 10 SNPs before a robust conclusion can be drawn. This means some of our results could not be analysed using this method, if there were not enough SNPs consisting the instrument we constructed.

Our design assumes that the samples used to define the glioma SNPs and the SNPs used to proxy the risk factors are illustrative of the same population, in terms of being comparable in ethnicity, age and sex distribution [83]. If these assumptions did not hold true, then the size of the association between each risk factor and glioma may be biased but such a violation will not necessarily increase the probability of mistakenly inferring a causal association if one does not exist [84]. Additionally, spurious associations may arise because of population stratification [85]. As all our instruments for potential risk factors and outcome (glioma) were collated from European populations, population stratification is made less likely but residual stratification remains a possibility. Not all the risk factors examined had enough power to detect the causal estimate, to increase the power would require additional instruments and larger sample sizes.

Furthermore, MR aims to draw causality between exposure and outcome, but the previous point of horizontal pleiotropy remains and can confound such conclusions. To this end, MR is a hypothesis-generating method that can guide further research into underlying mechanisms that drive the relationships identified by the analysis. Whilst some of our results show a robust association between the risk factor of interest and glioma risk, further studies with differing sources of confounding are required to accurately conclude causality. These can include in vitro and in vivo experiments, prospective cohort studies and other such epidemiological studies.

## Conclusion

In summary, by implementing a comprehensive MR study design, we corroborated previous MR studies suggesting a causal link between longer telomere length and increased glioma risk. Our findings suggested a positive association with genetic liability to allergic diseases. The findings from our study warrant further research to uncover the mechanism that implicates telomere length, allergic disease and metabolic-related traits (particularly, childhood extreme obesity and LDLc and triglyceride levels) in glioma onset.

## Supplementary information

**Additional file 1.**

**Additional file 2.**

**Additional file 3.**

**Additional file 4 Additional file, Table 1:** Details of instrumented risk factors found from the systematic search of the literature. **Additional file, Table 2**: Power calculations for each instrumented risk factor. **Additional file, Table 3**: 25 studies from which the 36 risk factors were evidenced. **Additional file, Table 4**: Detailed results from sensitivity analyses for each association that met at least the suggestive *P* value threshold in the IVW analysis. “NA” means there were not enough instruments to conduct this test. “r2” for the Steiger test indicates variance explained and “direction” is given: true, if effect is from exposure to outcome; false, if the effect is from outcome to exposure. **Additional file, Table 5**: Polygenic Risk Score results for each risk factor and all glioma. **Additional file, Table 6**: Polygenic Risk Scores for risk factors and glioblastoma. **Additional file, Table 7**: Polygenic Risk Score results for risk factors and non-glioblastoma (low-grade glioma).

**Additional file 5.**

**Additional file 6.**

**Additional file 7.**

## Data Availability

Genotype data from the Glioma International case-control and University of Texas M. D. Anderson Cancer Centre study GWAS are available from the European Genome-phenome Archive under accession EGAD00010001657 (https://www.ebi.ac.uk/ega/dacs/EGAC00001001116).

## Abbreviations

CI: Confidence interval
GWAS: Genome-wide association study
IV: Instrumental variable
IVW: Inverse-variance weighting
WME: Weighted median estimator
MBE: Mode-based estimator
*MR*: Mendelian randomization
*OR*: Odds ratio
*SNP*: Single nucleotide polymorphism
SD: Standard deviation
RCT: Randomised controlled trial
GICC: Glioma International case-control study
MDA: University of Texas M.D. Anderson Cancer center
PRS: Polygenic risk scores
InSIDE: Instrument strength independent of direct effects
BADGERS: Biobank-wide Association Discovery using GEnetic Risk Scores
