# Supplementary figures and images for: Testing for causality between systematically identified risk factors and glioma: a Mendelian randomization study
Source: BMC Cancer. 2020 Jun 3;20:508. doi: 10.1186/s12885-020-06967-2 (PMC7268455; doi:10.1186/s12885-020-06967-2)

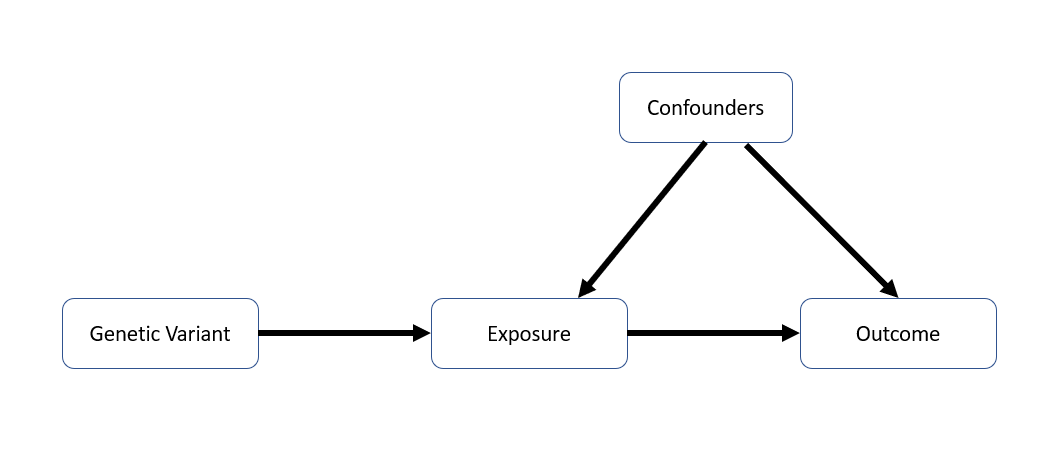

Supplement: Supplementary file 1 — Additional file 1. [file 12885_2020_6967_MOESM1_ESM.png]

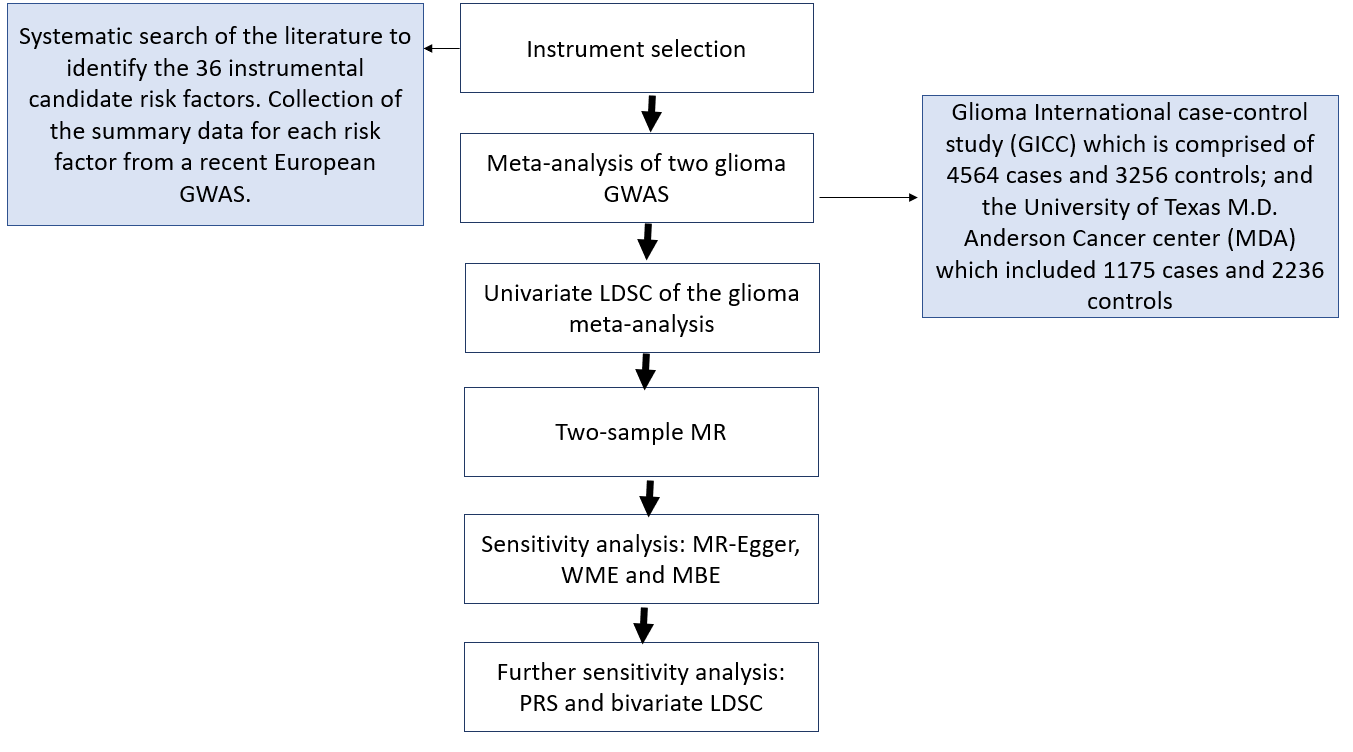

Supplement: Supplementary file 2 — Additional file 2. [file 12885_2020_6967_MOESM2_ESM.png]

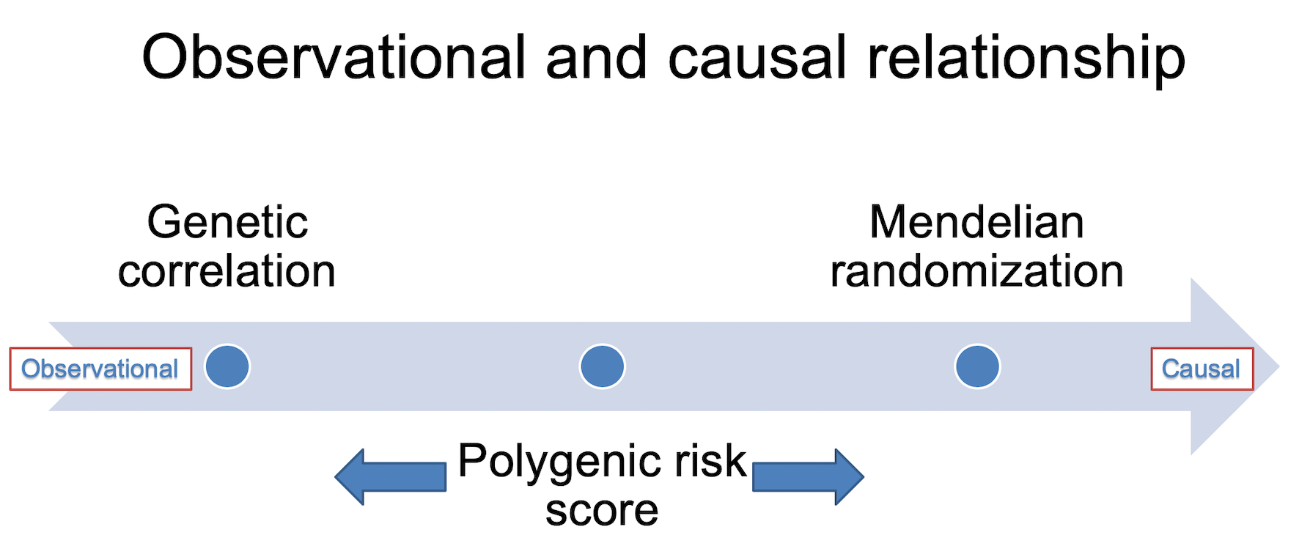

Supplement: Supplementary file 5 — Additional file 5. [file 12885_2020_6967_MOESM5_ESM.png]

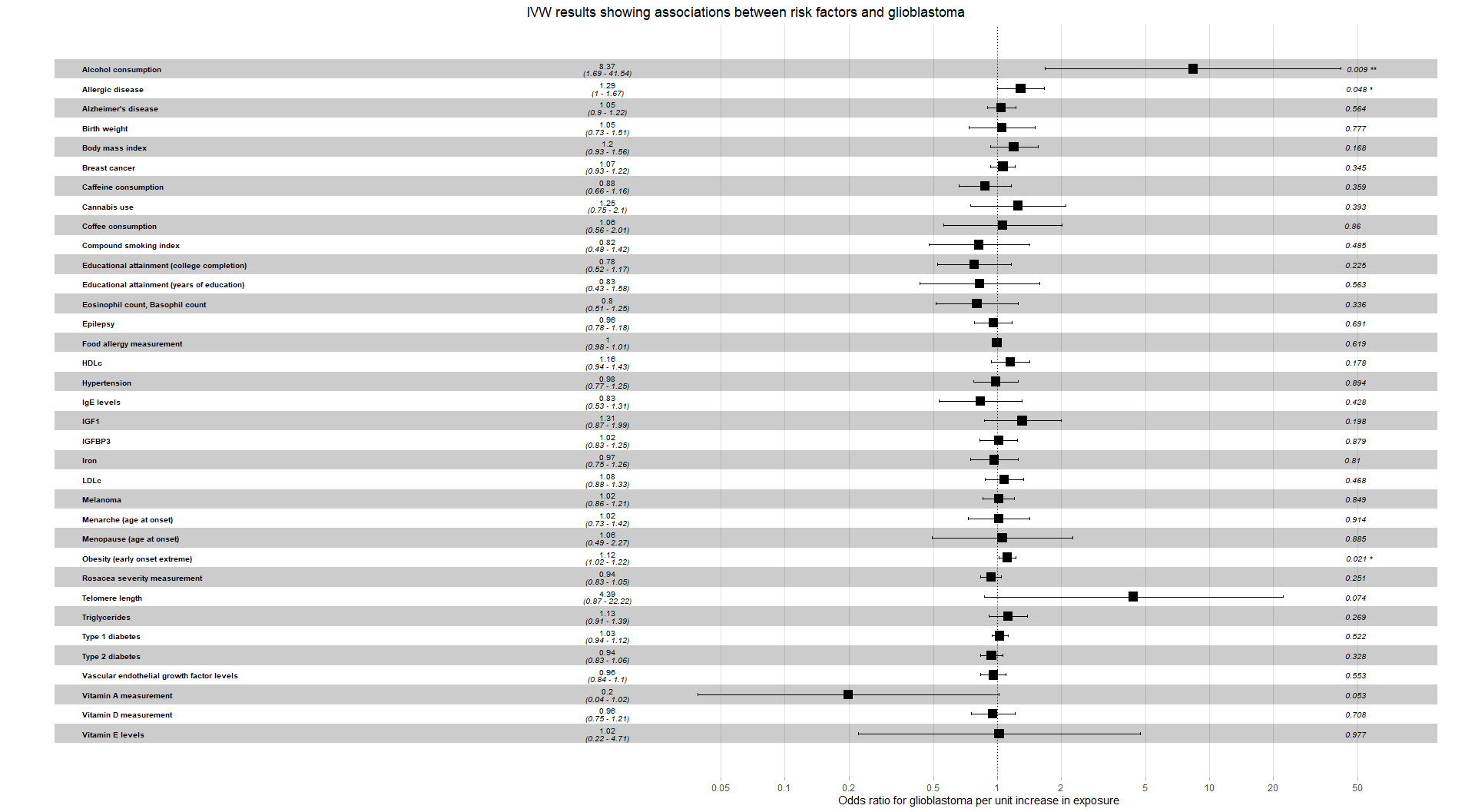

Supplement: Supplementary file 6 — Additional file 6. [file 12885_2020_6967_MOESM6_ESM.png]

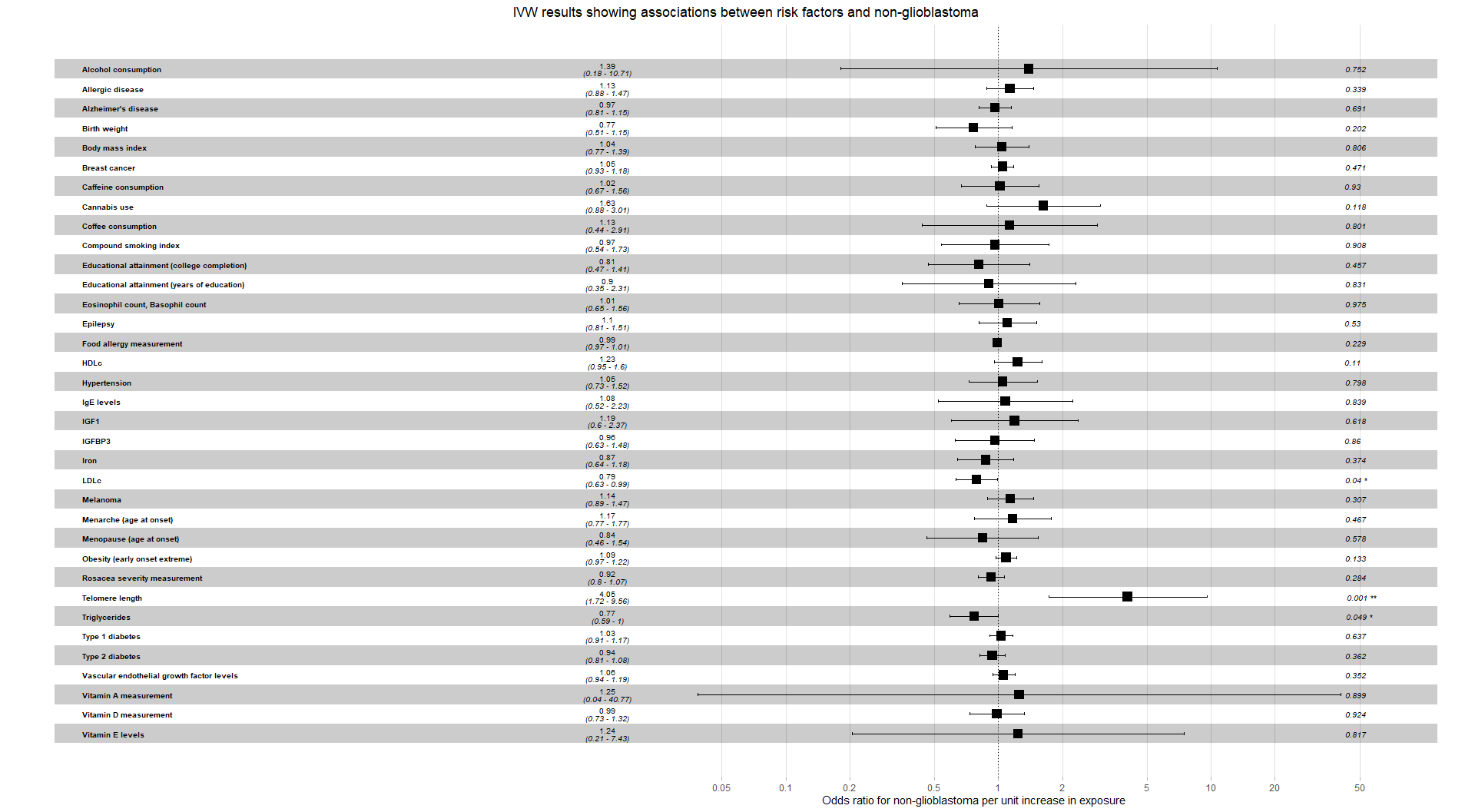

Supplement: Supplementary file 7 — Additional file 7. [file 12885_2020_6967_MOESM7_ESM.png]
