# Supplementary material for: Testing for causality between systematically identified risk factors and glioma: a Mendelian randomization study
Source: BMC Cancer. 2020 Jun 3;20:508. doi: 10.1186/s12885-020-06967-2 (PMC7268455; doi:10.1186/s12885-020-06967-2)
Supplement: Supplementary file 3 — Additional file 3. [file 12885_2020_6967_MOESM3_ESM.docx]

# Additional Note 1

We initially designed a search strategy to identify studies that report looking at ("risk" or a variety of exposures) AND (glioma or some form of glioma) AND (are systematic reviews). Secondly, we searched for primary studies that examined glioma risk.

Objective: To identify exposures that have been examined to determine whether they increase or decrease the risk of glioma.

Search strategy

Database: Ovid MEDLINE(R) and Epub Ahead of Print, In-Process & Other Non-Indexed Citations and Daily <1946 to October 23, 2018>

Search Strategy:

--------------------------------------------------------------------------------

1 RISK/ or RISK FACTORS/ or RISK ASSESSMENT/ (1014706)

2 risk?.ti. (428332)

3 or/1-2 (1157671)

4 exp Dietary Exposure/ (230)

5 exp Dietary Supplements/ (65089)

6 Environmental Exposure/ (68610)

7 Environmental Pollutants/ (33564)

8 (exposure or exposures).ab. /freq=3 (150975)

9 exp Electromagnetic Radiation/ or Radiation Exposure/ (312014)

10 Occupational Exposure/ (50817)

11 Maternal Exposure/ (7807)

12 Prenatal Exposure Delayed Effects/ (26172)

13 Paternal Exposure/ (959)

14 or/4-13 (647879)

15 exp Glioma/ (75734)

16 (glioma* or astrocytoma* or ependymoma* or oligodendroglioma* or glioblastoma* or ganglioglioma* or gliosarcoma*).ti,ab,kf. (83941)

17 Brain Neoplasms/ (102804)

18 ((brain or intracranial) adj (cancer? or neoplasm? or tumo?r)).ti,ab,kf. (30109)

19 or/15-18 (161379)

20 (3 or 14) and 19 (6426)

21 (systematic or structured or evidence or trials or studies).ti. and ((review or overview or look or examination or update* or summary).ti. or review.pt.) (168578)

22 (0266-4623 or 1469-493X or 1366-5278 or 1530-440X or 2046-4053).is. (17208)

23 meta-analysis.pt. or (meta-analys* or meta analys* or metaanalys* or meta synth* or meta-synth* or metasynth*).ti,ab,kf,hw. (164110)

24 ((systematic or meta) adj2 (analys* or review)).ti,kf. or ((systematic* or quantitativ* or methodologic*) adj5 (review* or overview*)).ti,ab,kf,sh. or (quantitativ$ adj5 synthesis$).ti,ab,kf,hw. (202947)

25 (integrative research review* or research integration).tw. or scoping review?.ti,kf. or (review.ti,kf,pt. and (trials as topic or studies as topic).hw.) or (evidence adj3 review*).ti,ab,kf. (174987)

26 review.pt. and ((medline or medlars or embase or pubmed or scisearch or psychinfo or psycinfo or psychlit or psyclit or cinahl or electronic database* or bibliographic database* or computeri#ed database* or online database* or pooling or pooled or mantel haenszel or peto or dersimonian or der simonian or fixed effect or ((hand adj2 search*) or (manual* adj2 search*))).tw,hw. or (retraction of publication or retracted publication).pt.) (137291)

27 or/21-26 (476874)

28 20 and 27 (438)

29 ((brain or intracranial) and (cancer? or neoplasm* or tumo?r)).ti. (12572)

30 cancer?.ti. (889225)

31 ((brain or intracranial) and (cancer? or neoplasm* or tumo?r)).ab,kf. (87722)

32 (glioma* or astrocytoma* or ependymoma* or oligodendroglioma* or glioblastoma* or ganglioglioma* or gliosarcoma*).ab,kf. (77234)

33 30 and (31 or 32) (12813)

34 29 or 33 (22213)

35 (3 or 14) and 27 and 34 (159)

36 28 or 35 (492)

Database: Ovid MEDLINE(R) and Epub Ahead of Print, In-Process & Other Non-Indexed Citations and Daily <1946 to October 31, 2018>

Search Strategy:

--------------------------------------------------------------------------------

1 Brain Neoplasms/ep, et, pc [Epidemiology, Etiology, Prevention & Control] (5447)

2 exp Glioma/ep, et, pc [Epidemiology, Etiology, Prevention & Control] (2608)

3 1 or 2 (6432)

4 exp odds ratio/ or proportional hazards models/ or exp risk/ or incidence/ (1288930)

5 exp Dietary Exposure/ (254)

6 exp Dietary Supplements/ (65265)

7 Environmental Exposure/ (68679)

8 Environmental Pollutants/ (33590)

9 exp Electromagnetic Radiation/ or Radiation Exposure/ (312311)

10 Occupational Exposure/ (50884)

11 Maternal Exposure/ or Paternal Exposure/ (8360)

12 Prenatal Exposure Delayed Effects/ (26209)

13 (exposure or exposures).ab. /freq=3 (151047)

14 or/4-13 (1877144)

15 3 and 14 (2036)

16 (glioma* or astrocytoma* or ependymoma* or oligodendroglioma* or glioblastoma* or ganglioglioma* or gliosarcoma*).ti. (53122)

17 (glioma or gliomas or astrocytoma or astrocytomas or ependymoma or ependymomas or oligodendroglioma or oligodendrogliomas or glioblastoma or glioblastomas or ganglioglioma or gangliogliomas or gliosarcoma or gliosarcomas).ab. /freq=3 (31313)

18 16 or 17 (58591)

19 (risk or risks).ab. /freq=3 (453104)

20 (risk or risks).ti. (425590)

21 19 or 20 (685558)

22 18 and 21 (986)

23 ((protective or protect against or protective against or risk? or risk factor* or cause* or aetiology or etiology) adj4 (glioma* or astrocytoma* or ependymoma* or oligodendroglioma* or glioblastoma* or ganglioglioma* or gliosarcoma*)).ti,ab. (1666)

24 (3 or 14 or 18 or 21) and 23 (1565)

25 ((protective or protect against or protective against or risk? or risk factor* or cause* or aetiology or etiology) adj2 (glioma* or astrocytoma* or ependymoma* or oligodendroglioma* or glioblastoma* or ganglioglioma* or gliosarcoma*)).ti,ab. (958)

26 15 or 22 or 24 or 25 (3578)

27 epidemiologic studies/ or follow-up studies/ or longitudinal studies/ or prospective studies/ or retrospective studies/ (1681804)

28 exp case control studies/ (950490)

29 exp cohort studies/ (1792765)

30 (control* adj2 (trial or study or studies)).ti,ab,kf. (326781)

31 (case control* or case series or (cohort adj (analys* or compar* or data or study or studies))).ti,ab,kf. (342843)

32 ((observational or followup or follow-up) adj (data or study or studies)).ti,ab,kf. (155721)

33 (longitudinal or retrospective or prospective).ti,ab,kf. (1100474)

34 (case control* or cohort? or follow-up or followup or longitudinal or prospective or retrospective or observational or population).ti. (592634)

35 (registry or registries).ti,ab,kf,hw. (141624)

36 (trial or study).ti,ab,kf. (7119324)

37 (case? adj4 control?).ab. (143788)

38 or/27-37 (8224376)

39 (systematic or structured or evidence or trials or studies).ti. and ((review or overview or look or examination or update* or summary).ti. or review.pt.) (168847)

40 (0266-4623 or 1469-493X or 1366-5278 or 1530-440X or 2046-4053).is. (17232)

41 meta-analysis.pt. or (meta-analys* or meta analys* or metaanalys* or meta synth* or meta-synth* or metasynth*).ti,ab,kf,hw. (164371)

42 ((systematic or meta) adj2 (analys* or review)).ti,kf. or ((systematic* or quantitativ* or methodologic*) adj5 (review* or overview*)).ti,ab,kf,sh. or (quantitativ$ adj5 synthesis$).ti,ab,kf,hw. (203270)

43 (integrative research review* or research integration).tw. or scoping review?.ti,kf. or (review.ti,kf,pt. and (trials as topic or studies as topic).hw.) or (evidence adj3 review*).ti,ab,kf. (175267)

44 review.pt. and ((medline or medlars or embase or pubmed or scisearch or psychinfo or psycinfo or psychlit or psyclit or cinahl or electronic database* or bibliographic database* or computeri#ed database* or online database* or pooling or pooled or mantel haenszel or peto or dersimonian or der simonian or fixed effect or ((hand adj2 search*) or (manual* adj2 search*))).tw,hw. or (retraction of publication or retracted publication).pt.) (137670)

45 or/39-44 (477526)

46 ("in data review" or in process or "pubmed not medline").st. (3159728)

47 26 and (38 or 45 or 46) (2653)

48 animals/ not humans/ (4477311)

49 47 not 48 (2607)

To ensure the same references were not screened twice, a duplicate screen was run with the results from both searches. The second search yield 2358 unique studies.

Inclusion and Exclusion criteria

Inclusion criteria: Studies that have examined the association between a non-genetic or epigenetic exposure and glioma in humans

Exclusion criteria:

• Studies which have examined genetic risk factors (e.g. SNPs, mutations)

• Studies which have examined epigenetic risk factors (e.g. DNA methylation)

• Studies which have examined RNA levels as risk factors

• Studies which didn’t link exposure with disease (e.g. descriptive studies looking at, for e.g. prevalence)

• Case-only studies (including case reports and case series)

• Animal or in vitro studies

• Studies which looked at the outcomes of glioma treatment (including surgery)

• Narrative reviews

• Family studies (Familial risk)

• Mendelian randomisation studies

• Missing full text

• Study was not published in English

• Childhood glioma (>50% of glioma cases occurred in patients <18y of age/eqv)

• Studies that do not specify glioma

# Additional Note 2

Oral contraceptive, Hormone replacement therapy, Menopause, Breast cancer, Age at menarche, Age at first birth, Magnetic fields, Mobile phones, Smoking, Blood eosinophil counts, Alcohol consumption, Body-mass index, Height, Farming, Aspirin, Tylenol, Non-steroidal anti-inflammatory drugs, COX2-inhibitors, Ibuprofen, Type 1 diabetes, Melanoma, Unprocessed red meat intake, Consumption of processed meats, Total meat intake, Hair dye, Type 2 diabetes, Obesity, Cannabis use, Underweight, Chicken pox, History of any allergy, Respiratory allergies, Age of diagnosis of respiratory allergies, Animal/insect allergies, Food allergies, Medication allergies, Soap/cosmetics allergies, Breastfeeding, Parity, Radiation, Coffee, Tea, Synthetic rubber, Pulp and paper, Magnetic field, Lead, Pesticides, Asthma, Epilepsy, Family history of cancer, Atopic dermatitis, Hay fever, Arthritis, Alzheimer’s, Medical intervention (cochlear implant patients), Waist circumference, Body somatotypes, Birth weight, Pre-diagnostic weight loss, Glucose-6-phosphate dehydrogenase deficiency, Education, Income, Marital status, Anti-depressants, Blood group, Handedness, Metabolites, Diltiazem, Verapamil, Amiodarone, Digoxin, Years since last birth, Insulin-like growth factor-1, Insulin-like growth factor binding protein-3, CCL22, Telomere length, Nitrate, Vitamin C, Vitamin E, Nitrite, Fruit and vegetable consumption, Statin use, Rosacea, Cadmium, Chromium, Iron, Nickel, Welding, Vascular endothelial growth factor levels, Beta-catenin, Leukaemia inhibitory factor, sIL10RB, Birth length, Head circumference, Head injury, Hypertension, Triglycerides, Hysterectomy, Construction, Transport, Chemical, Electrical, Vegetables, Cured and non-cured meats, Fish, Eggs/dairy, Green tea, Physical activity, Antioxidant index, Carotenoid, Matairesinol, Secoisolariciresinol, Coumestrol, Meningitis, low-density lipoprotein cholesterol, high-density lipoprotein cholesterol, Vitamin D, Dietary polyunsaturated fatty acid, positive penicillin skin tests (PenSTs), Immunoglobulin E levels, Long-term antihistamine use, Dentist, Artist, Methylene chloride, Intra cranial volume, Toenail selenium, Electromagnetic field, Insecticides, Herbicides, Glycosides, Lynch Syndrome, Immunoglobulin G antibodies, Petrochemical exposure, Neurocysticercosis, Polio vaccination, Antiepileptic drug, Dental X-ray, Aviation, Estimated glomerular filtration rate and Chlorinated water.
